# Supplementary material for: Genetic comparisons of interleukin-17 reveal a framework for complex signaling evolution
Source: bioRxiv. 2026 Apr 14:2026.04.13.718218. Preprint. [Version 1] doi: 10.64898/2026.04.13.718218 (PMC13105070; doi:10.64898/2026.04.13.718218)
Supplement: Supplement 3 [file media-3.zip › Supplemental files for paper/ERC/IL17xIL17_comparisons/IL17RC_IL17REL.pdf]

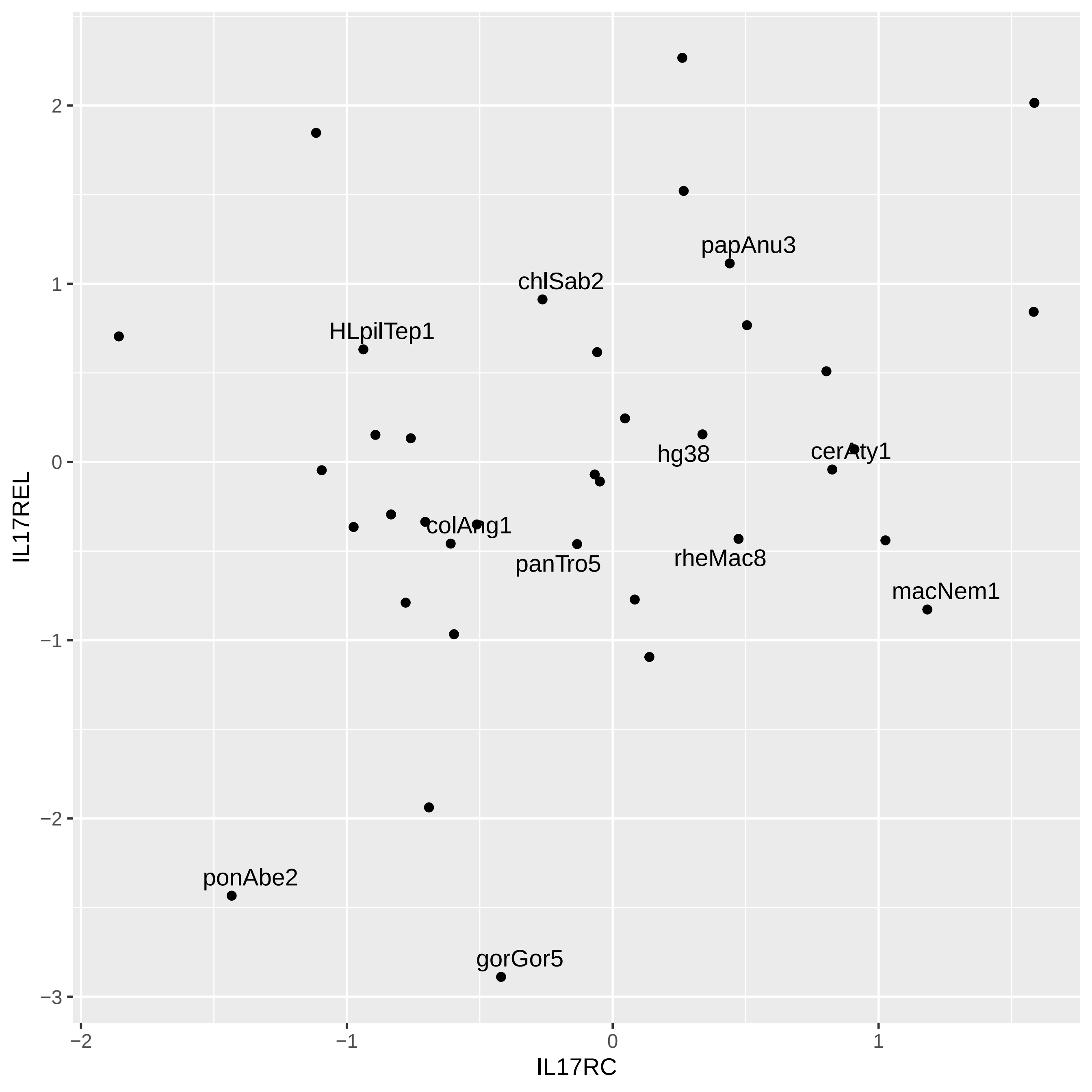

IL17REL
IL17RC
2
1
0
−1
−2
−3
−2
−1
0
1
papAnu3
chlSab2
HLpilTep1
hg38
cerAty1
colAng1
panTro5
rheMac8
macNem1
ponAbe2
gorGor5
